# Cellular Heterogeneity and Plasticity of Skin Epithelial Cells in Wound Healing and Tumorigenesis

**DOI:** 10.1007/s12015-021-10295-8

**Published:** 2022-02-10

**Authors:** Jingru Wang, Jia He, Meishu Zhu, Yan Han, Ronghua Yang, Hongwei Liu, Xuejuan Xu, Xiaodong Chen

**Affiliations:** 1 Department of Plastic Surgery, The First Affiliated Hospital of Jinan University, Guangzhou, China; 2 Department of Burn Surgery, First People’s Hospital of Foshan, Foshan, China; 3 School of Pharmaceutical Sciences (Shenzhen), Sun Yat-sen University, Guangzhou, China; 4 Department of Burn and Plastic Surgery, Second People’s Hospital of Shenzhen, First Affiliated Hospital of Shenzhen University Health Science Center, Shenzhen, China; 5 The Yonghe Medical Group Limited Company, George Town, Cayman Islands; 6 Endocrinology Department, First People’s Hospital of Foshan, Foshan, China

**Keywords:** Heterogeneity, Plasticity, Epidermal stem cells, Wound healing, Tumorigenesis

## Abstract

**Abstract:**

Cellular differentiation, the fundamental hallmark of cells, plays a critical role in homeostasis. And stem cells not only regulate the process where embryonic stem cells develop into a complete organism, but also replace ageing or damaged cells by proliferation, differentiation and migration. In characterizing distinct subpopulations of skin epithelial cells, stem cells show large heterogeneity and plasticity for homeostasis, wound healing and tumorigenesis. Epithelial stem cells and committed progenitors replenish each other or by themselves owing to the remarkable plasticity and heterogeneity of epidermal cells under certain circumstance. The development of new assay methods, including single-cell RNA sequence, lineage tracing assay, intravital microscopy systems and photon-ablation assay, highlight the plasticity of epidermal stem cells in response to injure and tumorigenesis. However, the critical mechanisms and key factors that regulate cellular plasticity still need for further exploration. In this review, we discuss the recent insights about the heterogeneity and plasticity of epithelial stem cells in homeostasis, wound healing and skin tumorigenesis. Understanding how stem cells collaborate together to repair injury and initiate tumor will offer new solutions for relevant diseases.

**Graphical Abstract:**

Schematic abstract of cellular heterogeneity and plasticity of skin epithelial cells in wound healing and tumorigenesis

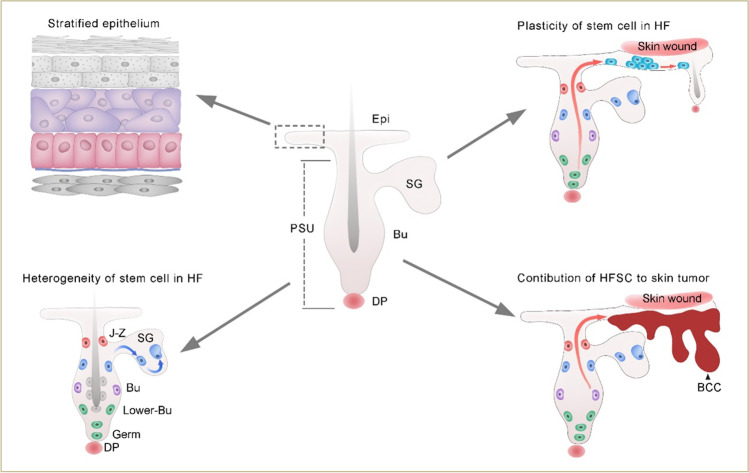

## Introduction

Adult stem cells are characterized by multipotency, asymmetric division, and have the capacity for self-renewal. Adult stem cells exist in kinds of organs, such as blood, small intestine, epidermis, and others. Adult stem cell, committed progenitors and differentiated functional cells co-create cellular heterogeneity [1, 2]. The plasticity of adult stem cells shows the lineage determination of a differentiating stem cell is flexible in respones to microenvironmental regenerative cues [3]. In characterizing distinct subpopulations of skin epithelial cells, adult stem cells show remarkable heterogeneity and plasticity under a particular physiological or pathological condition [4, 5].

The epidermis functions as a protective barrier for the body and is crucial in protecting against hostile environment and retaining bodily fluids inside [6]. The interfollicular epidermis (IFE) is the major component of the epidermis, and it prevents microbial intrusion and external environmental insults. The IFE is a stratified epithelium in which proliferating cells are anchored to the basement membrane closest to the dermis [7, 8]. The pilosebaceous unit (PSU) is a prominent structure associated with the IFE. The PSU exists important functions within the epidermis that are mediated via its major components, including infundibulum, isthmus, sebaceous glands and the hair follicle [9]. The epidermis and its appendages developed from multipotent embryonic progenitor keratinocytes, which receive cues from their environment that instruct them to commit to a certain differentiation programme and generate a stratified epidermis, hair follicles or sebaceous glands [10].The epidermis and its appendages are a highly dynamic tissue that undergoes constant turnover during normal steady-state conditions [11, 12]. Multiple stem cell populations residing in autonomously maintained compartments facilitate this task [4, 13]. Epidermal stem cells (EpSCs) locate in different niches have their own markers and functions [14]. IFESCs locate in the basal layer of the IFE and replenish the basal layer; IFESCs express high levels of β1 and α6 integrins, LRIG1 and MCSP [15, 16]. Hair follicle SCs (HFSCs) reside in bulge and possess specific bulge markers, including CD34, LGR5, Sox9 and so on; HFSCs maintain the hair lineages [14, 17]. Additionally, sebaceous gland SCs give rise to differentiated sebocytes [18]. EpSCs have been studied for possible proliferative potential since the 1970 s to form stratified squamous epithelium with more advanced keratinisation by epithelial cells from human skin biopsies [19]. Subsequently, cellular heterogeneity defined by marker expression, cell division rate and ultrastructure, has been studied both in IFE and PSU [4].

The epidermis and its appendages receive daily assaults, such as harmful ultraviolet radiation from the sun, scratches and wounds. It confronts these attacks by undergoing continual self-renewal to repair damaged tissue and replace aged cells [10]. In response to injury, different stem cell populations exhibit functionally flexibility. Upon injury, both HFSCs and IFESCs near the wound site mobilize toward it for reepithelializing the wound bed and restoring the barrier [20–22]. Once HFSCs and IFESCs are recruited to the IFE, they progressively lose their initial identity and are reprogrammed to an IFE fate [20]. However, the molecular mechanisms underlying this plasticity are still not clear. In a compared chromatin profiles of injured IFE and homeostatic HFSCs and IFESCs, both IFESC (Klf5) and HFSC (Sox9) transcription factors are enriched in the open chromatin regions of injured IFE, which indicate injured IFE get a hybrid signature [23]. Beside, HFSCs mobilize and re-epithelialize the injured skin, most HFSC transcription factors will be silence but Sox9, which remains active until the wound heals [24]. Moreover, several studies have reported differentiated suprabasal epidermal cells are able to revert back to a stem cell state when injured [25, 26].It is noteworthy that the wound size can influence cellular plasticity. De novo hair follicle formation is present in large wounds rather than small wound. Lineage tracing demonstrated these de novo hair follicles do not originate from HFSCs but from IFE cells [27].

The most common type of skin cancer is basal cell carcinoma (BCC), which arises from the deregulated Hedgehog signaling [28]. BCCs can arise from multiple stem cell populations, including the hair follicle bulge and the IFE [29]. The originating cells of BCC can influence the subtypes of BCC that develops, and can also affect the likelihood that a tumor will form [30]. Whereas inappropriate activation Hedgehog signaling is associated with the formation of BCC, deregulated WNT signaling is detected in different epidermal tumor types. In humans, activating mutations in β-catenin have been found in pilomatricomas and trichofolliculomas [31].

The stem cells located in IFE and PSU exhibit extensive heterogeneity under homeostasis condition, and highly plasticity during wound healing and tumorigenesis. In this Review, we discuss stem cell feature and behavior during normal tissue homeostasis, wound healing and tumor development within the epidermis. We provide an up-to-date view of the stem cells in epidermis, encompassing the heterogeneity and plasticity of multiple discrete stem cell populations in PSU that are strongly influenced by external cues to maintain their identity and function.

## Cellular Heterogeneity and Plasticity of Epidermis

### The Specific Populations of Interfollicular Epidermis and Hair Follicles

**Fig. 1:**
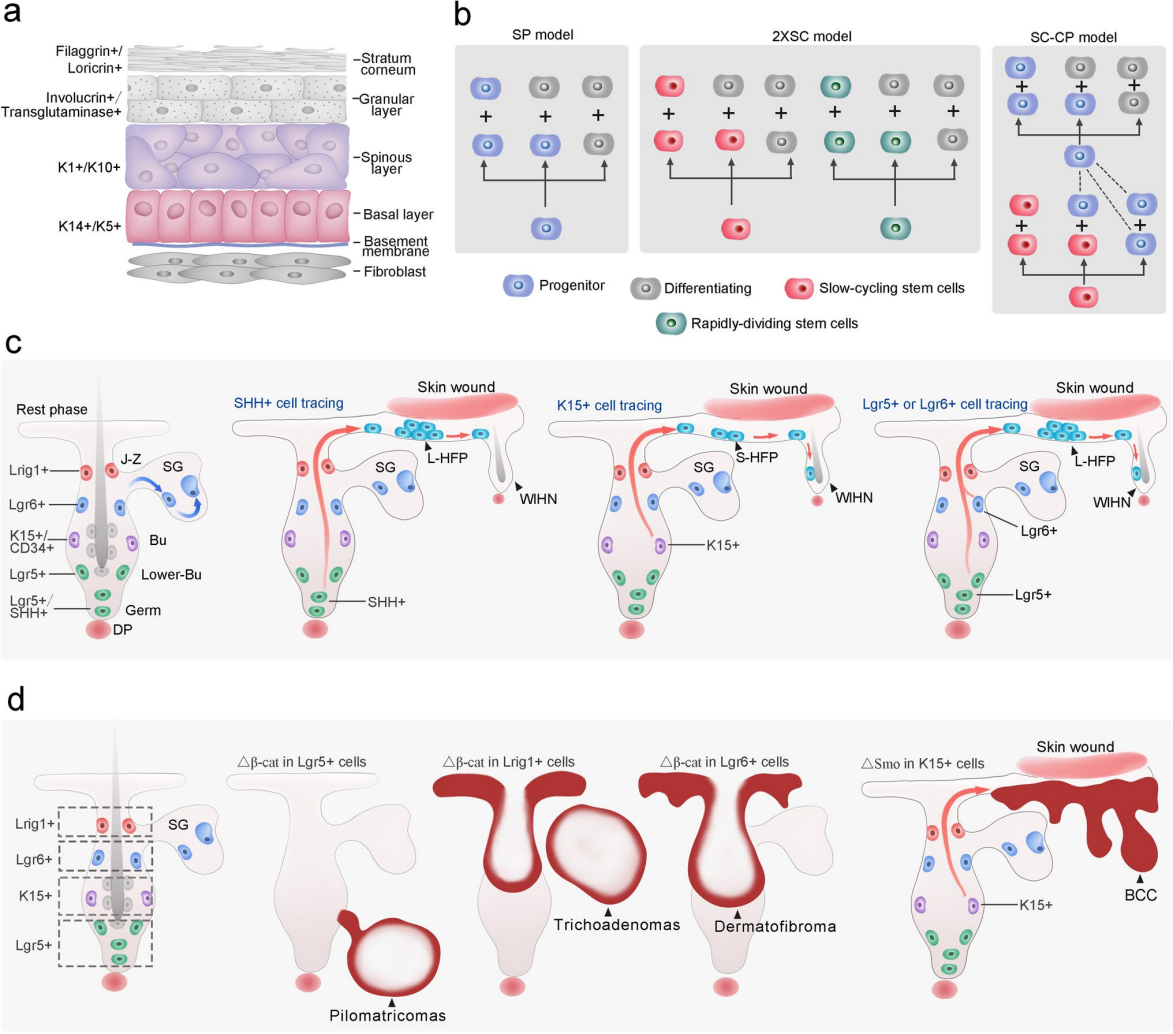
**a** The epidermis is a stratified structure that is composed of the basal cell layer and the underneath basement membrane, spinous layers, granular layers and stratum corneum layers. **b** Three stochastic and incompatible cell-proliferation models have been used to illustrate epidermal self-renewal, including the single-progenitor (SP) model, two stem-cell (2xSC) model and stem cell-committed progenitor (SC-CP) model. **c** The heterogeneity of stem cells in hair follicle. The hair follicle stem cells in junctional zone (J-Z) express Lrig1. Stem cell in isthmus express Lgr6. Stem cell in bulge (Bu) area express CD34 and Keratin15 (K15). Stem cells in lower bulge and hair germ express Lgr5, and Shh is uniquely expressed in hair germ. Hair follicle stem cells showed excellent plasticity during skin wounding. Shh+ hair germ cells could contribute to form new epidermis, and the progeny of Shh+ cells could survive in new epidermis over 16 weeks post wounding. K15+ bulge cells could contribute to both new epidermis and hair follicle within the wound. While only a minority of K15+ cell progeny remained at the 50th day post wound (Short-live hair follicle cell progeny, S-HFP). Both the Lgr5+ cells and Lgr6+ cells progeny contribute to from new epidermis and their progeny could been detected in new hair follicle within wound. And the progeny of both Lgr5+ and Lgr6+ follicular cells could been detected after 100 days post wound (Long-live hair follicle cell progeny, L-HFP). SG, sebaceous gland; DP, dermal papilla. **d** Compartmentalization of hair follicle stem cells underlies different responses to oncogene and skin tumor heterogeneity. Oncogenic β-catenin expression in Lgr5+ cells led to formation of pilomatricomas, while Lrig1+ cells formed trichoadenomas and Lgr6+ cells formed dermatofibromas. Expression of an activated form of *Smoothened* (SmoM2), a mediator of Hedgehog (Hh) signaling, by K15+ bulge does not produce basal cell carcinomas (BCCs). However, wounding induces these cells from the follicle to the wound site, where downstream Hh signal transduction is derepressed, and giving rise to superficial BCC-like tumors. △β-cat, active form of β-catenin; △Smo, active form of Smothened

Skin is the largest organ in the body, which includes epidermis, underlying dermis and adipose layer [32]. The epidermis consists of interfollicular epidermis (IFE) and its derivative appendages, including hair follicles, sebaceous glands, sweat glands [33]. The multi-tasking stratified epidermis, as the skin barrier, plays a pivotal role in protecting organism against environment assaults, preventing the water loss, regulating the temperature and so on [34]. The keratinized stratified epidermis is composed of four layers, including basal layer (Keratin5/Keratin14^+^), spinous layer (Keratin1/Keratin10^+^), granular layer (Involucrin^+^, Transglutaminase^+^) and cornified layer (Filaggrin^+^, Loricrin^+^) [35] (Fig. 1a). The renewal of epidermis depends on the cell division in the deepest basal layers [36, 37]. Afterwards the cells move up to the spinous layers, granular layers and cornified layers successively, and with the changing of cell morphological characteristics from lower layers to upper layers [38]. Under normal conditions, epidermal stem cells differentiated into keratinocytes that are shed from epithelial surface constantly. And the ageing or damaged keratinocytes would be replaced by new cells at a certain rate [39]. Single-cell RNA sequence (scRNA-seq) analysis uncovered the transcriptional diversity and complexity in the skin epithelial cells and highlighted how epidermal cells were tuned to ensure homeostasis. The first single-cell mapping of mouse 1,422 epidermal cells identified 25 populations, including 5 subpopulations from IFE, and revealed that differentiation and spatial signatures of these populations could assure tissue homeostasis [40]. Human studies of 92,889 epidermal cells from 9 normal and 3 inflamed skin samples showed stereotyped keratinocyte subpopulations exhibited a distinct composition at different anatomic sites. In keratinocytes, 12 % of transcripts differentially expressed between stereotyped patterns, revealing undescribed gene expression programs governing epidermal homeostasis [41]. Through scRNA-seq of follicle-enriched fractions of human skin, the transcriptional signatures of 23 primary cell clusters and lineage trajectory of epidermal and follicular cell progenitors were characterized [42].

In addition, hair follicles also possess simple structure and different stem cell populations, which provide an excellent model to study cellular heterogeneity and plasticity. Hair follicles harbor a permanent bulge region and undergo a cyclic regeneration through anagen, catagen and telogen [43, 44]. Hair follicles consists of hair germ (Gli1^+^, Lgr5^+^), bulge (Krt15^+^, CD34^+^, Lgr5^+^(lower portion)), isthmus (Lgr6^+^, Blimp1^+^), sebaceous gland (Lgr6^+^, Blimp1^+^) and infundibulum from bottom to top. The junctional zone (JZ) refers to the sebaceous gland and upper isthmus that is contiguous to infundibulum [4]. The location and distribution of hair follicle stem cells are showed in Fig. 1c. Furthermore, scRNA-seq and single-molecule RNA FISH analysis landscaped a systematic molecular atlas and identified 56 subpopulations associated with epithelial and stromal cells during telogen and anagen in hair follicles, unveiling the transcriptional dynamics of hair follicles during homeostasis [45].

### The Heterogeneity of Interfollicular Epidermis and Hair Follicles

The epidermal stem cells (EpSCs) and progenitor cells show excellent heterogeneity during homeostasis and wound healing. The stratum basale contains two mainly cell populations, including long-lived stem cells and ‘transit amplifying’ progenitor cells, which raises the research boom for decades [16, 46–48].

Three stochastic and incompatible cell-proliferation models have been used to illustrate epidermal self-renewal, including the single-progenitor (SP) model, stem cell-committed progenitor (SC-CP) model and two stem-cell (2xSC) model (Fig. 1b) [49]. SP model is the simplest model to describe the cellular proliferation in the basal layer.

In SP model, either inducible genetic labelling assay or clone-size distribution analysis supported that the progenitors were capable to produce proliferating cells and differentiating cells equally during homeostasis. However, during wound healing, the progenitor cells were biased to generate proliferating daughters excessively rather than slow cycling stem cells, suggesting that the progenitor cells played a key role in tissue repair [46, 49, 50]. The stem cell-committed progenitor (SC-CP) model showed that slow cycling stem cells produced stem cells and progenitors, and the latter preferred to differentiate so the renewal of stem cells is essential for epidermal maintenance [16, 51]. Mascré et al. suggested that during the wound healing, the stem cells contributed to reconstruct epidermis but committed progenitors played a limited role [16]. In addition, 2xSC model shows another way to expound homeostasis. Basal epidermal cells contain two types of stem cells, called slow-cycling stem cells and rapidly-dividing stem cells and both of them participate in maintaining homeostasis. The slow-cycling stem cells were known as label-retaining cells (LRCs) while rapidly-dividing stem cells were called non-LRC cells. The LRCs and non-LRCs represented two different stem cell populations and exhibited different patterns of proliferation and differentiation via lineage tracing assay. During homeostasis, two types of stem cells had distinct territories while they replenished each other during skin wounding [48].

Of note, scRNA-seq analysis unveiled the transcriptional states of human and murine epidermis in detail, enhancing our understanding of heterogenous epidermis in homeostasis and wound healing [52–54]. It was found that the basal cells harbor two distinct subpopulations transcriptionally, calling IFE B1 and IFE B2. Both of them express *Krt14/Krt5* at high levels while IFE B1 subpopulations also highly express *Avpi1*, *Krt16*, *Thbs1*, *Bhlhe40* additionally, further illustrating the diversity of interfollicular epidermis in mice [40]. Furthermore, in applying scRNA-seq with other related analysis, it was reported that the mouse epidermal basal cells exhibited four basal cell states, including Col17a^+^/Trp63^+^,Fos^+^, Cdkn1a^+^ and Mki67^+^ states during homeostasis. The Col 17a^+^/Trp63^+^ state stood for proliferative states while Fos^+^, Cdkn1a^+^, Mki67^+^ states represented non-proliferative states. These four states were spatially partitioned and showed highly plasticity during wound re-epithelialization [53]. ScRNA-seq analysis of human IFE uncovered the four specific stem cell populations spatially in the basal stem cells, supporting the multi-stem cells transition model rather than SP model during homeostasis [54]. More specific genes or key regulatory mechanisms during wound healing remains to be illustrated in further studies.

Hair follicles harbor extensive heterogeneity in pilosebaceous unit, which contains infundibulum, isthmus, sebaceous gland, bulge and hair germ [4]. Using K14-H2BGFP mice to mark label-retaining cells (LRCs),Elaine Fuchs et al. proved that the majority of LRCs were CD34^+^ bulge stem cells [17, 55]. And recent data demonstrated that the periphery of the placode basal layer was the source of bulge stem cells [56]. However, it was found that quiescent bulge stem cells do not directly give rise to ‘transit amplifying’ (TA) cells, namely matrix cells, but generate Lgr5^+^ progenitor cells located in the hair germ. Subsequently the Lgr5^+^ populations divide into TA cells, which further differentiate and migrate to form inner root sheath (IRS) and hair shaft [57–59]. Furthermore, Lgr5+ cells, which refers to a population of lower bulge and hair germ in telogen and outer root sheath (ORS) in anagen, could give rise to all linage cells during hair cycle and form new hair follicles. Importantly, Lgr5^+^ lineage tracing cells could repopulate the compartments like CD34^+^ bulge stem cells [60]. Also, the study determined that the cultured CD34^+^ bulge cells could repopulate the entire hair follicles *ex vivo* after transplantation [17, 55, 61]. Cultured CD34^+^ bulge cells promoted new hair formation at the site of transplantation and generated cells in epidermis, ORS, IRS and sebaceous glands appeared to possess functional SCs [55]. In addition, cultured bulge-derived stem cells which containing about 70 % CD34^+^ cells differentiated into vascular endothelial cells, epidermal cells, and ORS cells of HF after transplantion [61]. Compared with other SCs, cultured CD34^+^ bulge cells have differentially expressed genes mainly related with cell adhesion/extracellular matrix, cytoskeleton. This suggests CD34^+^ bulge cells maybe more suitable to differentiate and migrate during wound repair or upon activation of the hair cycle [55].

Lgr6^+^ cells,which refer to the majority of isthmus, sebaceous gland and interfollicular epidermis, play an important role in maintaining homeostasis of upper hair follicles. The Lgr6+ progenitor cells renewed themselves by population asymmetry within each compartment of upper hair follicles, ensuring homeostasis. However, the transcriptomics elucidated that Lgr6^+^ cells had no difference with Lgr6^−^ cells in gene expression signature [62]. Lgr6^+^ expression is interrelated with nerve endings/Schwann cells and the Schwann cells were degenerated following the disappear of Lgr6^+^ cells [63].

Extensive heterogeneity of human and mouse hair follicles is revealed by several scRNA-seq analysis. It landscaped the molecular signatures and heterogeneous states of different hair follicle populations [40, 42]. In general, recent researches open new avenues for us to deepen understanding of hair follicle heterogeneities and push the field of stem cell biology forward.

### The Plasticity of Interfollicular Epidermis

Epidermal stem cells and progenitor cells play a central role during the wound healing and tissue regeneration [64]. As early as the 20th century, how the epidermal cells migrate to the wound site have remained great controversies [65], which mainly contains two re-epithelialization mechanisms, a ‘wavefront’ model and a ‘leapfrog’ model [66]. A ‘wavefront’ model exhibited that basal progenitors could migrate to wound bed and reconstruct epidermis [67] while a ‘leapfrog’ model illustrated that the differentiated cells could leave base membrane and de-differentiate to promote the re-organization of epidermis [68, 69]. The epithelial cells of skin have long been deemed to lack of the de-differentiated capacity, and recent researches reinforce the ‘wavefront’ model [70, 71]. Unexpectedly, the skin epithelial cells have unparalleled plasticity in response to injury. And Giacomo Donati et al. opened up new avenues for de-differentiation of committed cells in the epidermis. Correspondingly, they illustrated that differentiated Gata6^+^ sebaceous duct cells involved proliferation and migration during wound healing and reverted to reattach the base membrane. The process promoted the regeneration of IFE, which reinforcing the re-epithelialization mechanism of a ‘leapfrog’ model. Subsequently, their lab also showed that Blimp1^+^ cells exhibited similar de-differentiation potential in response to injury [72]. Of note, these works resolve the debates that whether differentiated cells could contribute to epidermal repair and promote for further investigation about the mechanism of de-differentiation in skin repair [72]. Noteworthy, it was found that Col17a+ populations in basal layer and SP1 populations in spinous layer showed faster dynamics in wound site via RNA velocity analysis. It revealed the possibility of interconversion between basal cells and spinor cells, which extend the plasticity of interfollicular epidermis [72].

Additionally, it was reported that de-differentiation may be relevant to inflammatory response, since the crosstalk between immunity and stem cells made contributions to reestablish epidermis during wound healing [73]. Wound healing begins with an inflammatory phase when macrophages and neutrophils are the first involved. Secreted inflammatory mediators regulate the migration, proliferation, and differentiation of epidermal stem cells. IL1 produced by keratinocytes, neutrophils, and macrophages regulates epidermal stem cells via the caspase 8 signaling pathway [74]. Absent Aim2 and its downstream effector, caspase 1 and IL-1β, enhances the migration of epidermal stem cells and accelerates epithelialization [75]. TNF-α induces AKT phosphorylation in epidermal stem cells, and AKT signals activate downstream β-catenin signalling [76]. Besides, the TNFR1-dependent and -independent apoptosis affects the epidermal differentiation [77]. Of note, Th/1/Th2 cytokine modulation of CXCR2 expression correlates with proliferation of epidermal keratinocytes [78].

Although the phenomenon of de-differentiation had been reported, some problems are needed for further study conclusively and directly. For instance, whether other differentiated populations could de-differentiate into epidermal stem cells? Whether the de-differentiated-derived cells play a key role for lineage differentiation and wound healing? What’s the specific mechanisms to regulate de-differentiation and how to raise efficiency of de-differentiation? Further lineage tracing studies in combination with single cell transcriptome will provide more evidence to cellular differentiaiton in vivo and explore its mechanism conclusively. To summarize, the de-differentiation in skin has shed light on a novel strategy to understand how the epidermal cells restore homeostasis based on the cellular plasticity.

### The Plasticity of Hair Follicles

The diversity of hair follicle subpopulations has been wildly studied via the isolated assay, yet in the context of injury, the hair follicle stem cells (HFSCs) exhibited remarkable plasticity. The technical developments such as scRNA-seq analysis, genetic lineage tracing systems, intravital microscopy systems and photon-ablation assay have been deepened our understanding of cellular plasticity in the hair follicles [52, 79].

During the homeostasis, hair follicle niches are often compartmentalized as well as heterogeneous. However, when the niches are damaged, HFSCs would been re-shuffled anatomically and functionally, which could replenish all epidermal cells [20, 72, 80–82]. Specifically, when the epidermis suffered injury, the Krt15^+^ bulge stem cells served as ‘transient amplifying’ cells to replace the missing epidermis rapidly, but over time, Krt15^+^ progeny was eliminated and made limit contribution to wound-induced hair follicle neogenesis (WIHN) (Krt15^+^ progeny persisted in less than 3 % nascent hair follicle in the WIHN) [20, 27, 73, 83]. Moreover, Vered Levy et al. increased the understanding of the re-epithelialization during injury. In vivo lineage tracing assay utilizing Shh-cre and Krt15-cre mice illustrated that both Krt15+ and Shh^+^ progeny could replace epidermal stem cells after wounding, however, Shh^+^ progeny remained in the epidermis for months rather than K15^+^ progeny [20, 22]. In addition, Zhu et al. showed that isolated CD34^+^ skin cells could regenerate hair follicle and sebaceous *ex vivo* [84]. Intriguingly, other stem cells in hair follicle, such as Gli1^+^ [85], Sox9^+^ [86], Lgr5^+^ and Lgr6^+^ cells [52], played a role in wound repair and their progenies remained in epidermis for a period of time upon wounding (Fig. 1c), in which Lgr6^+^ cell progeny existed for a long time when participating the re-epithelialization, coupled with contributing to the formation of new hair follicles in wound. And over 100 days post wounding (dpw), Lgr6^+^ progeny accounted for 10 % of nascent hair follicles during WIHN [87]. Huang et al. discovered that Lgr6^+^ epidermal stem cells exhibited a pronounced response during the initial stages of wound re-epithelialization and interacted with nerves essentially to regulate their fate during wound healing [79]. Lough et al. also determined that Lgr6+ cells promoted re-epithelialization and hair follicle regeneration after transplantation into wound bed of nude mice [88]. What’s more, wang et al. reported that Lgr5^+^ progeny contributed to regenerate epidermis and WIHN, and 40 % nascent hair follicle contained Lgr5^+^ progeny [76]. Lineage tracing revealed that Lgr5^+^ progeny migrated out of the HFs and repopulated the infundibular area; Additionally, keratinocytes originating from Lgr5+ progeny integrated into the the newly formed wound epidermis [82, 89]. Intriguingly, according to single-cell transcriptomics, comparative works between Lgr5+ and Lgr6+ progeny cells were performed to illustrate their molecular heterogeneity and transcriptomic convergence in the context of wound environment. During injury, Lgr5+ lineage tracing cells elevated the gene expression that related to IFE-like signatures but decreasing the bulge related genes within 1 day. However, Lgr6^+^ progeny exhibited distinct transcriptional states that had already received the wound signals in the normal healthy skin [52]. Sixia Huang et al. elicited that Lgr6^+^ cells contributed to re-epithelialization depend on the perineural stem cell niche by transgenic-ablation assay [79] and Gli1^+^ cells showed similar results [85].

Conversely, recent studies had suggested that when the bulge or hair germ region were ablated by laser, they wound be repopulated to recover the niche. In addition, laser ablation assay suggested that the hair germ and bulge cells were not essential for hair follicle regeneration [90]. Intriguingly, both the bulge stem cells and hair germ cells could interconvert into each other after ablation. Besides, the populations of junctional zone would repopulate bulge cells after laser ablation [90, 91]. Moreover, after ablation of Lgr5^+^ hair germ stem cells, CD34^+^ bulge stem cells recover the Lgr5+ cells via inflammatory response and Wnt signaling [92]. Besides, IL-1, IL-17, and TNF, promote hair follicle neogenesis and epithelialization in wound healing [74, 76, 93]. Recent study showed Treg-cell control of a CXCL5-IL-17 inflammatory axis promoted migration and differentiation of Lgr5^+^ hair follicle stem cells in epithelialization [94]. Intriguingly, although some advanced technologies have been used for the study, the regulatory mechanisms involved the plasticity is a yet-unaddressed question and needs for further investigation.

## Cellular Plasticity and Related Oncogenes in Skin Tumorigenesis

### Oncogenic β-catenin in Skin Benign Tumorigenesis

Benign follicular tumors in skin share heterogenous characteristics, which show similar portions in normal hair follicles histologically. Thus, the classification of benign follicular tumors is based on the microscopic similarities between the normal hair follicles and neoplasm. The benign follicular tumors are heterogenous, including trichoadenoma, trichoblastoma, pilomatricoma [95], trichofolliculoma, etc [96]. Several benign follicular tumors are induced by the continuous activation of oncogenic β-catenin. Recent studies revealed heterogeneities of follicular stem cells during tumorigenesis, as the activation of β-catenin in different follicular populations gave rise to heterogenous benign tumors. Continuous activation of β-catenin in Krt14^+^ cells gave rise to follicular tumors that were similar to human trichofolliculomas and pilomatricomas [28, 97]. However, continuous activation of oncogenic β-catenin in Lgr5+ cells gave rise to pilomatricomas, while oncogenic β-catenin in Lgr6+ cells led to format dermatofibromas, and oncogenic β-catenin in Lrig1+ cells develop into trichoadenomas [98]. The activation β-catenin under the Krt15 promoter made no contributions to form neoplasm even with the upregulation of Wnt targeted genes, whereas continuous activation of β-catenin via ΔK5 promoters developed benign tumors [99]. Therefore, different hair follicle populations displayed different sensitivities upon activated β-catenin related benign tumors. However, human sebaceous tumors harbor Lef1 mutation, failing to bind the β-catenin site, which associated with inactive Wnt signals [100]. Furthermore, lacking the β-catenin binding site, the overexpression of DeltaNLef1 transgenic mice via Krt14 promoter gave rise to sebaceous tumors and exhibited sebaceous differentiation [101]. Additionally, knockout β-catenin in Krt14+ cells elicited the loss of cancer stem cells and regression of papillomas [102].

Conclusively, activating oncogenic β-catenin in different follicular stem cells develop into different types of tumors. Follicular tumors are occurred depending on the variant sensitivity to oncogenes of different hair follicle populations. And most of neoplasm exhibits activation of β-catenin, there’s some evidence that the tumors prefer to occur in the scalp since most of human scalp hair follicles are under anagen stage [96], which shows highly activation of β-catenin. The effect of oncogenes on different hair cycle stage is an interesting topic for further exploration.

### The Cellular Plasticity in BCC and SCC

The three main types of skin cancer are basal cell carcinoma (BCC), cutaneous squamous cell carcinoma (SCC) and melanoma, which occupy with the most of skin cancers and differ in many ways [103]. It has shown that BCC is occurred more widespread than SCC [104]. Both BCC and SCC are derived from the cells that residing in skin interfollicular epidermis and hair follicles, thus sharing the comparable origins [89, 105, 106]. Generally, BCC shows the mutation of *Patched (PTCH)* and *Smoothened (SMO)*, which relate to Hedgehog signaling pathway [4, 105], however, SCC is more likely to display the mutation of *p53* and *RAS* [33, 107].

In addition, emerging evidences supported that the committed cells could reserve as tumor-initiating cells via direct reprogramming [108]. For instance, during wound, differentiated cells in IFE promoted the initiation of papillomas following the activation of MEK [109]. And the differentiated cells could turn into tumor-initiating cells when the mutations of *H Ras* were induced under the control of K10 promoter, whose expression were restricted to IFE cells, and the formation of papillomas were observed without experimental second ‘hit’ after wounding [110]. Also, it was reported that BCC was induced by the activation of Smoothened mutant (SmoM2) in Krt14+ the basal cells, which conversed tumor initiating cells to embryonic HF progenitors and promoted the expression of embryonic genes via reprogramming [111]. Meanwhile, Wnt/β-catenin signaling pathway was activated speedily and the loss of β-catenin restrained the reprogramming and tumor initiation [111].

Therefore, cellular plasticity may play an important role in initiating BCC and SCC and further studies will be done to illustrate what types of cells could dedifferentiate to accelerate the tumor regrowth after therapy.

### The Heterogenous Origin of BCC and SCC

The constitutive activation of Hedgehog (HH) signaling develops BCCs whose are thought to be highly invasive under certain circumstances [112]. Several methods are used to establish BCC models in mouse epidermis, which achieved by losing of patch functions [113] or by motivating the expression of *Smoothened mutation* (*SmoM2*) that served as an oncogene [114], or through increasing the levels of Gli1 [115] and Gli2 [116].

Primarily, PTCH-induced BCCs shared diverse origins in mouse epidermis. The topical administration of retinoic acid in Krt6a-Cre:Ptch1^neo/neo^ mice, which showed that the Cre expression and ptch loss were restricted in IFE and infundibulum, developed the BCC formation, indicating that BCC was derived from IFE cells after PTCH deletion [117]. The bulge stem cells exhibited highly self-renewal capacity in the radiation-driven BCCs in ptc1 ^neo67/+^ mice, implicating that bulge stem cells may be the source of PTCH-induced BCCs [118]. Likewise, lineage tracing assay suggested that X-ray driven BCCs in Ptch1(+/-) mice could initiate from Krt15^+^ bulge stem cells [119].

Whereas, clonal analysis indicated that SmoM2-induced BCCs were derived from long-lived stem cells and/or progenitors that existing in interfollicular epidermis and the upper infundibulum [31, 51]. Intriguingly, Sunny Y et al. illustrated that during homeostasis, activating the expression of Smo via the Krt15 promoter does not form BCCs. However, Krt15^+^ tracing cells would be recruit to the wound side and these cells contributed to the initiation of BCCs, which made a connection between wound and cancer [120]. It was speculated that bulge stem cells migrated to IFE and got IFE fate, developing into the BCCs upon HH signaling. Of note, Ptch1+/-; K14CreER2; p53^fl/fl^ mice showed highly efficiency in initiating BCCs than Ptch1+/-; K15CrePR1; p53^fl/fl^ mice after administration of tamoxifen and RU-486 respectively [119].

In general, inactivated PTCH-induced BCCs originate preferentially from HFSCs [89, 119], while SmoM2-induced BCCs mainly arise in the IFE and upper infundibulum [31, 120]. Besides, increasing evidence unveiled the touch domes that resided in IFE is served as “hot spots” to promote the formation of BCCs [121, 122]. In addition, cutaneous nerve-derived Shh is required for the renewal of touch domes stem cells in the epidermis [121, 123].

Typically, the two-step methods are applied to develop SCC, in which mice are treated with DMBA for one time to serve as a tumor initiator, which induce the mutation of *H Ras*, subsequently, the mice are treated with TPA repeatedly to serve as a tumor promoter [33]. Whole-exome sequencing analysis uncovered that DMBA/TPA induced SCCs harbor recurrent mutations of *RAS* gene, such as *Hras*, *Kras*, *Rras2* and so on [124].

SCCs exhibit squamous differentiation morphologically, indicating that SCCs may drive from the cells IFE cells [125]. What’s more, DMBA treatment for one time and after one year, TPA administration still could initiate papillomas, suggesting that the mutation of *H Ras* originated from long-lived stem cells [126–128].

The *KRAS* mutations were triggered under the control of various promoters, such as Krt19, Krt15 and involucrin, leading to form papillomas [129], and furthermore, the loss of p53 in the context of *KRAS* mutation could develop into invasive SCCs [130]. And combined Ras mutation with NOTCH1/2 and CDKN1 mutants played a key role in triggering SCC formation [131]. These studies suggested that the formation of tumor initiating cells depend on the genetic lesion rather than on the targeted follicular stem cells [130]. Intriguingly, injury is implicated in developing epithelial tumors under the condition of activated *RAS* signals. Following wounding, the tumors were induced by activated SOS, RAS activator, via the Krt5 promoter [132]. Meanwhile, when the *HRAS* was activated by the promoter that activated in suprabasal IFE cells, tumors were restricted to the wound [110, 133], while the expression of HRAS using a K5 promoter, papilloma and SCCs were triggered spontaneously [134]. Additionally, mice were more sensitive to develop SCC when carrying gene mutation [135, 136]. The same mutations were occurred in different populations causing different types of SCCs, while the SCCs derived from IFE exhibited well-differentiated states and SCCs originated from hair follicle stem cells had the potential to promote tumor metastasis [137]. Thus, the development of epithelial tumors depends on both intrinsic oncogene insult and extrinsic environment. Cellular origin and it related oncogenes in contributing to different skin tumors are showed in Table 1.

**Table 1:** Cellular origin and it related oncogenes in contributing to different skin tumors

Tumor types	Mutant genes	The contributed cells for tumors	References
Trichofolliculomas/papillomatricomas	β-catenin	Krt 14^+^ cells	[6, 72]
Pilomatricomas	β-catenin	Lgr5^+^ cells	[73]
Dermatofibromas	β-catenin	Lgr6^+^ cells	[73]
Trichoadenomas	β-catenin	Lrig1^+^ cells	[73]
No tumor	β-catenin	Krt15^+^ cells	[74]
Sebaceous tumors	Lef1	Krt14^+^ cells	[75, 76]
Basal cell carcinoma(BCC)	Patched	IFE cells/bulge stem cells(major contribution)	[80, 93–95]
BCC	Smoothened	IFE cells	[9, 96]
BCC	Smoothened (in the context of wound)	Krt15^+^ bulge stem cells	[96]
Papillomas	KRAS	Krt19^+^, Krt15^+^ and Involucrin^+^ cells	[105]
Invasive squamous cell carcinoma (SCC)	P53 and KRAS	--	[106]
SCC (DMBA-TPA method)	HRAS	Long-lived stem cells	[102–104]
Papilloma and SCC	HRAS	Krt5^+^ cells	[110]

## Conclusion

Over several decades, a number of models have been proposed to explain epidermal maintenance. These models range from the existence of a single common multipotent stem cell population to no apparent stem cells at all. Stem cell hierarchy models adapted from the hematopoietic system have been used to explain tissue maintenance in most tissues. However, such models are not necessarily suitable for cell turnover in a spatially restricted environment such as the epithelia. Recent evidence provides a more comprehensive view which support multiple discrete stem cell populations with restricted lineage potential under homeostatic conditions serve to maintain specific compartments within the epidermis. The compartmentalization model was originally proposed within the epithelium of the mammary gland, where the luminal and basal cell compartments are maintained as independent entities [138]. The molecular feature and function of different stem cells in different compartment with PSU are welly established, while the interaction and relationship between these heterogeneous stem cells, during both homeostasis and tissue damage, still need further exploration. The further interpretation of hair follicle stem cell properties, also their interaction with microenvironment, will be helpful for developing new strategies to treat hair follicle related disease, such as androgenic alopecia and alopecia areata.

A better understanding of how the microenvironment direct stem cell dynamics and tissue compartmentalization will be important for detecting the specific factors that control stem cell property and behavior. For example, interactions between the dermis and epidermis in skin are integral for homeostasis and for the proper expression of stem cell markers within the PSU [63, 85]. However, the complexity of epidermal stem cells interaction with their niche is only starting to be revealed. Recent evidence suggests that the cells in dermis is highly heterogeneous and contains multiple distinct lineages [139]. Dermal stem cells, mainly dermal papilla or skin-derived precursors, are recruited for hair follicle formation during re-epithelialisation [139–141]. Dermal transcriptional repressor Blimp1 is a key mediator of epidermal TGFβ and Wnt/β-catenin signaling to regulate hair follicle formation and growth [142].

Subsequently, sc-RNA seq revealed differential BMP and WNT signaling established compartmentalization of epithelial-mesenchymal micro-niches [143]. Myofibroblasts and their microenvironment are key factors for maintaining skin homeostasis. Although myofibroblasts have a limited role in the re-epithelialization process, they can promote matrix proteins synthesis to induce the faster wound contraction [144–146]. Future explorations are desired and which will shed light on the reciprocal relationship between the cells in epidermis and dermis that control stem cell characteristics and behavior, and how skin tissue compartmentalization relates to health and disease.

Plasticity is the hallmark of epidermal stem cells, even embryonic epithelial cells are sensitive to the mesenchyme to which they are exposed. When contacted with mesenchyme tissue from chick wing, epidermis from the leg produces feathers, by contrast, mesenchyme from leg can prompt wing epidermis to make scales. The special features of cellular plasticity seem to be lost in most cells of majority of tissues as development proceeds, but stem cells of the skin seem retain this valuable potential. If bulge stem cells exposed to corneal mesenchyme and produce cornea, they could be used to treat certain types of blindness. Other possible uses for bulge stem cells might be in treating chronic ulcers or hair disorders. While the lose of hair follicle regenerating potential hair follicle stem cells during culturing impede their clinical application to hair disorder. However, the potential of cultured basal epidermal keratinocytes has already been realized to treat burns patients [147]. Thus, the level of plasticity afforded epidermal stem cells is becoming increasingly important as their valuable potential in regenerative medicine continue to be explored.

The stem cells in IFE and hair follicles exhibit extensive heterogeneity and plasticity in homeostasis, wound healing and tumorigenesis, which provides an excellent model to study stem cell biology for decades. New technological development including scRNA-seq and ATAC seq deepen our understanding of heterogeneity and plasticity of stem cells. Numerous studies have unveiled the complexity of the skin epidermis in different states. Sc-RNA seq analysis will landscape more detail classification of the heterogeneity in IFE, hair follicles and tumorigenesis, and open new avenues for dynamic stem cells behaviors.

Cells are capable to change their fate through lineage plasticity, including dedifferentiation and interconversion, whereas, how these cells collaborate to restore homeostasis? And what are the specific mechanisms to regulate the plasticity in skin epithelial cells? In addition, it’s clear that, scRNA-seq analysis showed similar characteristics in transcriptome and chromatin accessibility between the cells in wound and cancer, but how the cells orchestrate the process of wound healing or cancer initiation? Overall,reciprocal relationships between cellular states and cellular types in diverse skin epithelial cells remain for further investigation.

## Data Availability

All data generated or analyzed during this study are included in this published article.

## Abbreviations

IFE: interfollicular epidermis
scRNA-seq: single-cell RNA sequence
EpSCs: epidermal stem cells
IRS: inner root sheath
ORS: outer root sheath
HFSCs: hair follicle stem cells
WIHN: wound-induced hair follicle neogenesis
BCC: basal cell carcinoma
SCC: squamous cell carcinoma
PSU: pilosebaceous unit
JZ: junctional zone
SP: single-progenitor
SC-CP: stem cell-committed progenitor
2xSC: two stem-cell
LRCs: label-retaining cells
TA: transit amplifying
S-HFP: Short-live hair follicle cell progeny
L-HFP: Long-live hair follicle cell progeny
SG: sebaceous gland
DP: dermal papilla

## References

[1] Tang, D. G. (2012,). Understanding cancer stem cell heterogeneity and plasticity. *Cell Research*, *22*(3), 457–472 10.1038/cr.2012.13 PMC3292302 22357481

[2] Simons, B. D., & Clevers, H. (2011). Strategies for homeostatic stem cell self-renewal in adult tissues. *Cell, 145*(6), 851-862 10.1016/j.cell.2011.05.033 21663791

[3] Wagers, A. J. & Weissman, I. L. (2004). Plasticity of adult stem cells. *Cell*, *116*(5), 639–648 10.1016/s0092-8674(04)00208-9 15006347

[4] Schepeler T, Page ME, Jensen KB (2014). Heterogeneity and plasticity of epidermal stem cells. Development (Cambridge, England).

[5] Rognoni E, Watt FM (2018). Skin cell heterogeneity in development, wound healing, and cancer. Thends in Cell Biology.

[6] Fuchs, E., & Byrne, C. (1994). The epidermis: rising to the surface. *Current Opinion in Genetics & Development*, *4*(5), 725–736 10.1016/0959-437x(94)90140-x 7531523

[7] Plikus MV, Gay DL, Treffeisen E, Wang A, Supapannachart RJ, Cotsarelis G (2012). Epithelial stem cells and implications for wound repair. Seminars in Cell and Developmental Biology.

[8] Sada, A., Jacob, F., Leung, E., Wang, S., White, B. S., Shalloway, D., & Tumbar, T. (2016). Defining the cellular lineage hierarchy in the inter-follicular epidermis of adult skin. *Nature Cell Biology*, *18*(6), 619–631 10.1038/ncb3359 PMC4884151 27183471

[9] Ghazizadeh S, Taichman LB (2001). Multiple classes of stem cells in cutaneous epithelium: a lineage analysis of adult mouse skin. The EMBO Journal.

[10] Fuchs E (2007). Scratching the surface of skin development. Nature.

[11] Blanpain, C., & Fuchs, E. (2009). Epidermal homeostasis: a balancing act of stem cells in the skin. *Nature Reviews Molecular Cell Biology, 10*(3, 207–217 10.1038/nrm2636 PMC2760218 19209183

[12] Nassar, D., & Blanpain, C. (2012). Epidermal development and homeostasis. *Seminars in Cell & Developmental Biology*, *23*(8), 883 10.1016/j.semcdb.2012.09.005 23018016

[13] Flora, P., & Ezhkova, E. (2020). Regulatory mechanisms governing epidermal stem cell function during development and homeostasis. *Development,**147*(22), dev194100 10.1242/dev.194100 PMC7687856 33191273

[14] Yang, R., Liu, F., Wang, J., Chen, X., Xie, J., & Xiong, K. (2019). Epidermal stem cells in wound healing and their clinical applications. *Stem Cell Research & Therapy*, *10*(1), 229 10.1186/s13287-019-1312-z PMC6664527 31358069

[15] Jones, P. H., & Watt, F. M. (1993). Separation of human epidermal stem cells from transit amplifying cells on the basis of differences in integrin function and expression. *Cell*, *73*(4), 713-724 10.1016/0092-8674(93)90251-k 8500165

[16] Mascré, G., Dekoninck, S., Drogat, B., Youssef, K. K., Broheé, S., Sotiropoulou, P. A. … Blanpain, C. (2012). Distinct contribution of stem and progenitor cells to epidermal maintenance. *Nature*, *489*(7415), 257–262 10.1038/nature11393 22940863

[17] Blanpain C, Lowry WE, Geoghegan A, Polak L, Fuchs E (2004). Self-renewal, multipotency, and the existence of two cell populations within an epithelial stem cell niche. Cell.

[18] Veniaminova, N. A., Grachtchouk, M., Doane, O. J., Peterson, J. K., Quigley, D. A., & Lull, M. V., Pyrozhenko DV et al. (2019). Niche-specific factors dynamically regulate sebaceous gland stem cells in the skin. *Developmental Cell, 51*(3), 326-340 10.1016/j.devcel.2019.08.015 PMC6832854 31564613

[19] Rheinwald, J. G., & Green, H. (1975). Serial cultivation of strains of human epidermal keratinocytes: the formation of keratinizing colonies from single cells. *Cell,**6*, 331-343 10.1016/s0092-8674(75)80001-8 1052771

[20] Ito M, Liu Y, Yang Z, Nguyen J, Liang F, Morris RJ, Cotsarelis G (2005). Stem cells in the hair follicle bulge contribute to wound repair but not to homeostasis of the epidermis. Nature Medicine.

[21] Jensen KB, Collins CA, Nascimento E, Tan DW, Frye M, Itami S, Watt FM (2009). Lrig1 expression defines a distinct multipotent stem cell population in mammalian epidermis. Cell Stem Cell.

[22] Levy V, Lindon C, Zheng Y, Harfe BD, Morgan BA (2007). Epidermal stem cells arise from the hair follicle after wounding. FASEB Journal: Official Publication of the Federation of American Societies for Experimental Biology.

[23] Ge, Y., Gomez, N. C., Adam, R. C., Nikolova, M., Yang, H., Verma, A. … Fuchs, E. (2017). Stem cell lineage infidelity drives wound repair and cancer. *Cell, 169*(4), 636-650 10.1016/j.cell.2017.03.042 PMC5510746 28434617

[24] Adam, R. C., Yang, H., Rockowitz, S., Larsen, S. B., Nikolova, M., Oristian, D. S. … Asare, M. Kadaja A Zheng, D. (2015). Pioneer factors govern super-enhancer dynamics in stem cell plasticity and lineage choice. *Nature, 521*, 366–370 10.1038/nature14289 PMC4482136 25799994

[25] Fu X, Sun X, Li X, Sheng Z (2001). Dedifferentiation of epidermal cells to stem cells in vivo. Lancet.

[26] Mannik J, Alzayady K, Ghazizadeh S (2010). Regeneration of multilineage skin epithelia by differentiated keratinocytes. The Journal of Investigative Dermatology.

[27] Ito M, Yang Z, Andl T, Cui C, Kim N, Millar SE, Cotsarelis G (2007). Wnt-dependent de novo hair follicle regeneration in adult mouse skin after wounding. Nature.

[28] Gat U, DasGupta R, Degenstein L, Fuchs E (1998). De Novo hair follicle morphogenesis and hair tumors in mice expressing a truncated beta-catenin in skin. Cell.

[29] Hahn H, Wicking C, Zaphiropoulous PG, Gailani MR, Shanley S, Chidambaram A, Vorechovsky I, Holmberg E, Unden AB, Gillies S (1996). Mutations of the human homolog of Drosophila patched in the nevoid basal cell carcinoma syndrome. Cell.

[30] Grachtchouk M, Pero J, Yang SH, Ermilov AN, Michael LE, Wang A, Wilbert D, Patel RM, Ferris J, Diener J (2011). Basal cell carcinomas in mice arise from hair follicle stem cells and multiple epithelial progenitor populations. The Journal of Clinical Investigation.

[31] Youssef, K. K., Van Keymeulen, A., Lapouge, G., Beck, B., Michaux, C., Achouri, Y. … Blanpain, C. (2010). Identification of the cell lineage at the origin of basal cell carcinoma. *Nature Cell Biology*, *12*(3), 299–305 10.1038/ncb2031 20154679

[32] Gravitz L (2018). Skin. Nature.

[33] Arwert EN, Hoste E, Watt FM (2012). Epithelial stem cells, wound healing and cancer. Nature Reviews Cancer.

[34] Fujiwara H, Tsutsui K, Morita R (2018). Multi-tasking epidermal stem cells: Beyond epidermal maintenance. Development, Growth & Differentiation.

[35] Veltri A, Lang C, Lien WH (2018). Concise review: Wnt signaling pathways in skin development and epidermal stem cells. Stem Cells (Dayton, Ohio).

[36] Gonzales KAU, Fuchs E (2017). Skin and its regenerative powers: an alliance between stem cells and their niche. Developmental Cell.

[37] Liu S, Zhang H, Duan E (2013). Epidermal development in mammals: key regulators, signals from beneath, and stem cells. International Journal of Molecular Sciences.

[38] Guasch G, Blanpain C (2004). Defining the epithelial stem cell niche in skin. Medecine Sciences: M/S.

[39] Alonso L, Fuchs E (2003). Stem cells of the skin epithelium. Proceedings of the National Academy of Sciences of the United States of America.

[40] Joost, S., Zeisel, A., Jacob, T., Sun, X., La Manno, G., Lönnerberg, P. … Kasper, M. (2016). Single-cell transcriptomics reveals that differentiation and spatial signatures shape epidermal and hair follicle heterogeneity. *Cell Systems*, *3*(3), 221–237229 10.1016/j.cels.2016.08.010 PMC5052454 27641957

[41] Cheng JB, Sedgewick AJ, Finnegan AI, Harirchian P, Lee J, Kwon S, Fassett MS, Golovato J, Gray M, Ghadially R (2018). Transcriptional programming of normal and inflamed human epidermis at single-cell resolution. Cell Reports.

[42] Takahashi, R., Grzenda, A., Allison, T. F., Rawnsley, J., Balin, S. J., Sabri, S. … Lowry, W. E. (2020). Defining transcriptional signatures of human hair follicle cell states. *The Journal of Investigative Dermatology*, *140*(4), 764–773764 10.1016/j.jid.2019.07.726 PMC7093259 31676413

[43] Müller-Röver, S., Handjiski, B., van der Veen, C., Eichmüller, S., Foitzik, K., McKay, I. A. … Paus, R. (2001). A comprehensive guide for the accurate classification of murine hair follicles in distinct hair cycle stages. *The Journal of Investigative Dermatology*, *117*(1), 3–15 10.1046/j.0022-202x.2001.01377.x 11442744

[44] Wang X (2019). Stem cells in tissues, organoids, and cancers. Cellular and Molecular Life Sciences: CMLS.

[45] Joost, S., Annusver, K., Jacob, T., Sun, X., Dalessandri, T., Sivan, U. … Kasper, M. (2020). The molecular anatomy of mouse skin during hair growth and rest. *Cell Stem Cell*, *26*(3), 441–457447 10.1016/j.stem.2020.01.012 32109378

[46] Clayton E, Doupé DP, Klein AM, Winton DJ, Simons BD, Jones PH (2007). A single type of progenitor cell maintains normal epidermis. Nature.

[47] McKinley KL, Castillo-Azofeifa D, Klein OD (2020). Tools and concepts for interrogating and defining cellular identity. Cell Stem Cell.

[48] Sada A, Jacob F, Leung E, Wang S, White BS, Shalloway D, Tumbar T (2016). Defining the cellular lineage hierarchy in the interfollicular epidermis of adult skin. Nature Cell Biology.

[49] Piedrafita, G., Kostiou, V., Wabik, A., Colom, B., Fernandez-Antoran, D., Herms, A. … Jones, P. H. (2020). A single-progenitor model as the unifying paradigm of epidermal and esophageal epithelial maintenance in mice. *Nature Communications*, 11(1), 1429 10.1038/s41467-020-15258-0 PMC7080751 32188860

[50] Doupé DP, Alcolea MP, Roshan A, Zhang G, Klein AM, Simons BD, Jones PH (2012). A single progenitor population switches behavior to maintain and repair esophageal epithelium. Science (New York, NY).

[51] Sánchez-Danés A, Hannezo E, Larsimont JC, Liagre M, Youssef KK, Simons BD, Blanpain C (2016). Defining the clonal dynamics leading to mouse skin tumour initiation. Nature.

[52] Joost S, Jacob T, Sun X, Annusver K, La Manno G, Sur I, Kasper M (2018). Single-cell transcriptomics of traced epidermal and hair follicle stem cells reveals rapid adaptations during wound healing. Cell Reports.

[53] Haensel D, Jin S, Sun P, Cinco R, Dragan M, Nguyen Q, Cang Z, Gong Y, Vu R, MacLean AL (2020). Defining epidermal basal cell states during skin homeostasis and wound healing using single-cell transcriptomics. Cell Reports.

[54] Wang S, Drummond ML, Guerrero-Juarez CF, Tarapore E, MacLean AL, Stabell AR, Wu SC, Gutierrez G, That BT, Benavente CA (2020). Single cell transcriptomics of human epidermis identifies basal stem cell transition states. Nature Communications.

[55] Cotsarelis G, Sun TT, Lavker RM (1990). Label-retaining cells reside in the bulge area of pilosebaceous unit: implications for follicular stem cells, hair cycle, and skin carcinogenesis. Cell.

[56] Morita, R., Sanzen, N., Sasaki, H., Hayashi, T., Umeda, M., Yoshimura, M., Yamamoto, T., Shibata, T., Abe, T., Kiyonari, H., et al. (2021). Tracing the origin of hair follicle stem cells. *Nature*10.1038/s41586-021-03638-5 34108685

[57] Greco, V., Chen, T., Rendl, M., Schober, M., Pasolli, H. A., Stokes, N. … Fuchs, E. (2009). A two-step mechanism for stem cell activation during hair regeneration. *Cell Stem Cell*, *4*(2), 155–169 10.1016/j.stem.2008.12.009 PMC2668200 19200804

[58] Lee SA, Li KN, Tumbar T (2021). Stem cell-intrinsic mechanisms regulating adult hair follicle homeostasis. Experimental Dermatology.

[59] Zhang YV, Cheong J, Ciapurin N, McDermitt DJ, Tumbar T (2009). Distinct self-renewal and differentiation phases in the niche of infrequently dividing hair follicle stem cells. Cell Stem Cell.

[60] Jaks V, Barker N, Kasper M, van Es JH, Snippert HJ, Clevers H, Toftgård R (2008). Lgr5 marks cycling, yet long-lived, hair follicle stem cells. Nature Genetics.

[61] Cheng X, Yu Z, Song Y, Zhang Y, Du J, Su Y, Ma X (2020). Hair follicle bulge-derived stem cells promote tissue regeneration during skin expansion. Biomedicine & Pharmacotherapy.

[62] Füllgrabe A, Joost S, Are A, Jacob T, Sivan U, Haegebarth A, Linnarsson S, Simons BD, Clevers H, Toftgård R (2015). Dynamics of Lgr6^+^ progenitor cells in the hair follicle, sebaceous gland, and interfollicular epidermis. Stem Cell Reports.

[63] Liao XH, Nguyen H (2014). Epidermal expression of Lgr6 is dependent on nerve endings and Schwann cells. Experimental Dermatology.

[64] Donati G, Watt FM (2015). Stem cell heterogeneity and plasticity in epithelia. Cell Stem Cell.

[65] Headon D (2017). Reversing stratification during wound healing. Nature Cell Biology.

[66] Safferling, K., Sütterlin, T., Westphal, K., Ernst, C., Breuhahn, K., James, M. … Grabe, N. (2013). Wound healing revised: a novel reepithelialization mechanism revealed by in vitro and in silico models. *The Journal of Cell Biology*, *203*(4), 691–709 10.1083/jcb.201212020 PMC3840932 24385489

[67] Radice GP (1980). The spreading of epithelial cells during wound closure in Xenopus larvae. Developmental Biology.

[68] Krawczyk WS (1971). A pattern of epidermal cell migration during wound healing. The Journal of Cell Biology.

[69] Paladini RD, Takahashi K, Bravo NS, Coulombe PA (1996). Onset of re-epithelialization after skin injury correlates with a reorganization of keratin filaments in wound edge keratinocytes: defining a potential role for keratin 16. The Journal of Cell Biology.

[70] Aragona M, Dekoninck S, Rulands S, Lenglez S, Mascré G, Simons BD, Blanpain C (2017). Defining stem cell dynamics and migration during wound healing in mouse skin epidermis. Nature Communications.

[71] Park S, Greco V, Cockburn K (2016). Live imaging of stem cells: answering old questions and raising new ones. Current Opinion in Cell Biology.

[72] Donati G, Rognoni E, Hiratsuka T, Liakath-Ali K, Hoste E, Kar G, Kayikci M, Russell R, Kretzschmar K, Mulder KW (2017). Wounding induces dedifferentiation of epidermal Gata6(+) cells and acquisition of stem cell properties. Nature Cell Biology.

[73] Morgun EI, Vorotelyak EA (2020). Epidermal stem cells in hair follicle cycling and skin regeneration: a view from the perspective of inflammation. Frontiers in Cell and Developmental Biology.

[74] Lee, P., Gund, R., Dutta, A., Pincha, N., Rana, I., Ghosh, S. … Jamora, C. (2017). Stimulation of hair follicle stem cell proliferation through an IL-1 dependent activation of γδT-cells. *Elife*, *6*, e28875 10.7554/eLife.28875 PMC5714500 29199946

[75] Naik, S., Larsen, S. B., Gomez, N. C., Alaverdyan, K., Sendoel, A., Yuan, S. … Fuchs, E. (2017,). Inflammatory Memory Sensitizes Skin Epithelial Stem Cells to Tissue Damage. *Nature*, *550*(7677), 475–480 10.1038/nature24271 PMC5808576 29045388

[76] Wang X, Chen H, Tian R, Zhang Y, Drutskaya MS, Wang C, Ge J, Fan Z, Kong D, Wang X (2017). Macrophages induce AKT/β-catenin-dependent Lgr5(+) stem cell activation and hair follicle regeneration through TNF. Nature Communications.

[77] Piao, X., Miura, R., Miyake, S., Komazawa-Sakon, S., Koike, M., Shindo, R. … Nakano, H. (2019). Blockade of TNFR1-dependent and -independent cell death is crucial for normal epidermal differentiation. *Journal of Allergy and Clinical Immunology, 143*(1), 213-228 10.1016/j.jaci.2018.02.043 29596938

[78] Zaja-Milatovic, S., & Richmond, A. (2008). CXC chemokines and their receptors: a case for a significant biological role in cutaneous wound healing. *Histol Histopathol, 23*(11), 1399-1407 10.14670/hh-23.1399 PMC3140405 18785122

[79] Huang, S., Kuri, P., Aubert, Y., Brewster, M., Li, N., Farrelly, O., Rice, G., Bae, H., Prouty, S., Dentchev, T., et al. (2021). Lgr6 marks epidermal stem cells with a nerve-dependent role in wound re-epithelialization. *Cell Stem Cell*10.1016/j.stem.2021.05.007 PMC8528178 34102139

[80] Dekoninck S, Blanpain C (2019). Stem cell dynamics, migration and plasticity during wound healing. Nature Cell Biology.

[81] Levy V, Lindon C, Harfe BD, Morgan BA (2005). Distinct stem cell populations regenerate the follicle and interfollicular epidermis. Developmental Cell.

[82] Page ME, Lombard P, Ng F, Göttgens B, Jensen KB (2013). The epidermis comprises autonomous compartments maintained by distinct stem cell populations. Cell Stem Cell.

[83] Wier EM, Garza LA (2020). Through the lens of hair follicle neogenesis, a new focus on mechanisms of skin regeneration after wounding. Seminars in Cell & Developmental Biology.

[84] Zhu B, Nahmias Y, Yarmush ML, Murthy SK (2014). Microfluidic isolation of CD34-positive skin cells enables regeneration of hair and sebaceous glands in vivo. Stem Cells Translational Medicine.

[85] Brownell I, Guevara E, Bai CB, Loomis CA, Joyner AL (2011). Nerve-derived sonic hedgehog defines a niche for hair follicle stem cells capable of becoming epidermal stem cells. Cell Stem Cell.

[86] Nowak JA, Polak L, Pasolli HA, Fuchs E (2008). Hair follicle stem cells are specified and function in early skin morphogenesis. Cell Stem Cell.

[87] Snippert HJ, Haegebarth A, Kasper M, Jaks V, van Es JH, Barker N, van de Wetering M, van den Born M, Begthel H, Vries RG (2010). Lgr6 marks stem cells in the hair follicle that generate all cell lineages of the skin. Science (New York, NY).

[88] Lough, D. M., Yang, M., Blum, A., Reichensperger, J. D., Cosenza, N. M., Wetter, N. … Neumeister, M. W. (2014). Transplantation of the LGR6+ epithelial stem cell into full-thickness cutaneous wounds results in enhanced healing, nascent hair follicle development, and augmentation of angiogenic analytes. *Plastic and Reconstructive Surgery*, *133*(3), 579–590 10.1097/PRS.0000000000000075 24572851

[89] Kasper, M., Jaks, V., Are, A., Bergström, Ã., Schwäger, A., Svärd, J. … Toftgård, R. (2011). Wounding enhances epidermal tumorigenesis by recruiting hair follicle keratinocytes. *Proceedings of the National Academy of Sciences of the United States of America*, *108*(10), 4099–4104 10.1073/pnas.1014489108 PMC3054016 21321199

[90] Rompolas P, Mesa KR, Greco V (2013). Spatial organization within a niche as a determinant of stem-cell fate. Nature.

[91] Blanpain C, Fuchs E (2014). Stem cell plasticity. Plasticity of epithelial stem cells in tissue regeneration. Science (New York, NY).

[92] Hoeck, J. D., Biehs, B., Kurtova, A. V., Kljavin, N. M., de Sousa, E. M. F., Alicke, B. … de Sauvage, F. J. (2017). Stem cell plasticity enables hair regeneration following Lgr5(+) cell loss. *Nature Cell Biology*, *19*(6), 666–676 10.1038/ncb3535 28553937

[93] Xiao, T., Yan, Z., Xiao, S., & Xia, Y. (2020). Proinflammatory cytokines regulate epidermal stem cells in wound epithelialization. *Stem Cell Research & Therapy, 11*, 232 10.1186/s13287-020-01755-y PMC7291661 32527289

[94] Mathur, A. N., Zirak, B., Boothby, I. C., Tan, M., Cohen, J. N., Mauro, T. M. … Rosenblum, M. D. (2019). Treg-cell control of a CXCL5-IL-17 inflammatory axis promotes hair-follicle-stem-cell differentiation during skin-barrier repair. *Immunity, 50*(3), 655-667 10.1016/j.immuni.2019.02.013 PMC6507428 30893588

[95] Chan EF, Gat U, McNiff JM, Fuchs E (1999). A common human skin tumour is caused by activating mutations in beta-catenin. Nature Genetics.

[96] Tellechea, O., Cardoso, J. C., Reis, J. P., Ramos, L., Gameiro, A. R., Coutinho, I., & Baptista, A. P. (2015). : Benign follicular tumors. *Anais brasileiros de Dermatologia,**90*(6), 780-796; quiz 797-788 10.1590/abd1806-4841.20154114 PMC4689065 26734858

[97] Lo Celso C, Prowse DM, Watt FM (2004). Transient activation of beta-catenin signalling in adult mouse epidermis is sufficient to induce new hair follicles but continuous activation is required to maintain hair follicle tumours. Development (Cambridge, England).

[98] Kretzschmar K, Weber C, Driskell RR, Calonje E, Watt FM (2016). Compartmentalized epidermal activation of β-catenin differentially affects lineage reprogramming and underlies tumor heterogeneity. Cell Reports.

[99] Baker CM, Verstuyf A, Jensen KB, Watt FM (2010). Differential sensitivity of epidermal cell subpopulations to beta-catenin-induced ectopic hair follicle formation. Developmental Biology.

[100] Takeda, H., Lyle, S., Lazar, A. J., Zouboulis, C. C., Smyth, I., & Watt, F. M. (2006). Human sebaceous tumors harbor inactivating mutations in LEF1. *Nature Medicine,**12*(4), 395-397 10.1038/nm1386 16565724

[101] Niemann C, Owens DM, Hülsken J, Birchmeier W, Watt FM (2002). Expression of DeltaNLef1 in mouse epidermis results in differentiation of hair follicles into squamous epidermal cysts and formation of skin tumours. Development (Cambridge, England).

[102] Malanchi I, Peinado H, Kassen D, Hussenet T, Metzger D, Chambon P, Huber M, Hohl D, Cano A, Birchmeier W (2008). Cutaneous cancer stem cell maintenance is dependent on beta-catenin signalling. Nature.

[103] Lomas A, Leonardi-Bee J, Bath-Hextall F (2012). A systematic review of worldwide incidence of nonmelanoma skin cancer. The British Journal of Dermatology.

[104] Massand S, Neves RI (2019). Current basal and squamous cell skin cancer management. Plastic and Reconstructive Surgery.

[105] Kasper M, Jaks V, Hohl D, Toftgård R (2012). Basal cell carcinoma - molecular biology and potential new therapies. The Journal of Clinical Investigation.

[106] Lichtenberger BM, Kasper M (2021). Cellular heterogeneity and microenvironmental control of skin cancer. Journal of Internal Medicine.

[107] Burclaff, J., & Mills, J. C. (2018). Plasticity of differentiated cells in wound repair and tumorigenesis, part II: skin and intestine. *Disease Models & Mechanisms, **11*(9) 10.1242/dmm.035071 PMC6177008 30171151

[108] Song IY, Balmain A (2015). Cellular reprogramming in skin cancer. Seminars in Cancer Biology.

[109] Hobbs RM, Silva-Vargas V, Groves R, Watt FM (2004). Expression of activated MEK1 in differentiating epidermal cells is sufficient to generate hyperproliferative and inflammatory skin lesions. The Journal of Investigative Dermatology.

[110] Bailleul, B., Surani, M. A., White, S., Barton, S. C., Brown, K., Blessing, M. … Balmain, A. (1990). Skin hyperkeratosis and papilloma formation in transgenic mice expressing a ras oncogene from a suprabasal keratin promoter. *Cell*, *62*(4), 697–708 10.1016/0092-8674(90)90115-u 1696852

[111] Youssef KK, Lapouge G, Bouvrée K, Rorive S, Brohée S, Appelstein O, Larsimont JC, Sukumaran V, Van de Sande B, Pucci D (2012). Adult interfollicular tumour-initiating cells are reprogrammed into an embryonic hair follicle progenitor-like fate during basal cell carcinoma initiation. Nature Cell Biology.

[112] Epstein EH (2008). Basal cell carcinomas: attack of the hedgehog. Nature Reviews Cancer.

[113] Blanpain C (2013). Tracing the cellular origin of cancer. Nature Cell Biology.

[114] Xie J, Murone M, Luoh SM, Ryan A, Gu Q, Zhang C, Bonifas JM, Lam CW, Hynes M, Goddard A (1998). Activating Smoothened mutations in sporadic basal-cell carcinoma. Nature.

[115] Nilsson M, Undèn AB, Krause D, Malmqwist U, Raza K, Zaphiropoulos PG, Toftgård R (2000). Induction of basal cell carcinomas and trichoepitheliomas in mice overexpressing GLI-1. Proceedings of the National Academy of Sciences of the United States of America.

[116] Grachtchouk M, Mo R, Yu S, Zhang X, Sasaki H, Hui CC, Dlugosz AA (2000). Basal cell carcinomas in mice overexpressing Gli2 in skin. Nature Genetics.

[117] Adolphe C, Hetherington R, Ellis T, Wainwright B (2006). Patched1 functions as a gatekeeper by promoting cell cycle progression. Cancer Research.

[118] Mancuso, M., Leonardi, S., Tanori, M., Pasquali, E., Pierdomenico, M., Rebessi, S. … Saran, A. (2006). Hair cycle-dependent basal cell carcinoma tumorigenesis in Ptc1neo67/+ mice exposed to radiation. *Cancer Research*, *66*(13), 6606–6614 10.1158/0008-5472.CAN-05-3690 16818633

[119] Wang GY, Wang J, Mancianti ML, Epstein EH (2011). Basal cell carcinomas arise from hair follicle stem cells in Ptch1(+/-) mice. Cancer Cell.

[120] Wong SY, Reiter JF (2011). Wounding mobilizes hair follicle stem cells to form tumors. Proceedings of the National Academy of Sciences of the United States of America.

[121] Peterson, S. C., Eberl, M., Vagnozzi, A. N., Belkadi, A., Veniaminova, N. A., Verhaegen, M. E. … Wong, S. Y. (2015). Basal cell carcinoma preferentially arises from stem cells within hair follicle and mechanosensory niches. *Cell Stem Cell*, *16*(4), 400–412 10.1016/j.stem.2015.02.006 PMC4387376 25842978

[122] Sun, X., Are, A., Annusver, K., Sivan, U., Jacob, T., Dalessandri, T. … Kasper, M. (2020). Coordinated hedgehog signaling induces new hair follicles in adult skin. *eLife, **9* 10.7554/eLife.46756 PMC7077985 32178760

[123] Xiao Y, Thoresen DT, Williams JS, Wang C, Perna J, Petrova R, Brownell I (2015). Neural Hedgehog signaling maintains stem cell renewal in the sensory touch dome epithelium. Proceedings of the National Academy of Sciences of the United States of America.

[124] Nassar D, Latil M, Boeckx B, Lambrechts D, Blanpain C (2015). Genomic landscape of carcinogen-induced and genetically induced mouse skin squamous cell carcinoma. Nature Medicine.

[125] Owens DM, Watt FM (2003). Contribution of stem cells and differentiated cells to epidermal tumours. Nature Reviews Cancer.

[126] Morris RJ (2000). Keratinocyte stem cells: targets for cutaneous carcinogens. The Journal of Clinical Investigation.

[127] Morris RJ, Coulter K, Tryson K, Steinberg SR (1997). Evidence that cutaneous carcinogen-initiated epithelial cells from mice are quiescent rather than actively cycling. Cancer Research.

[128] Morris RJ, Fischer SM, Slaga TJ (1986). Evidence that a slowly cycling subpopulation of adult murine epidermal cells retains carcinogen. Cancer Research.

[129] White AC, Tran K, Khuu J, Dang C, Cui Y, Binder SW, Lowry WE (2011). Defining the origins of Ras/p53-mediated squamous cell carcinoma. Proceedings of the National Academy of Sciences of the United States of America.

[130] Lapouge G, Youssef KK, Vokaer B, Achouri Y, Michaux C, Sotiropoulou PA, Blanpain C (2011). Identifying the cellular origin of squamous skin tumors. Proceedings of the National Academy of Sciences of the United States of America.

[131] Dotto GP, Rustgi AK (2016). Squamous cell cancers. Cancer Cell.

[132] Sibilia, M., Fleischmann, A., Behrens, A., Stingl, L., Carroll, J., Watt, F. M. … Wagner, E. F. (2000). The EGF receptor provides an essential survival signal for SOS-dependent skin tumor development. *Cell*, *102*(2), 211–220 10.1016/s0092-8674(00)00026-x 10943841

[133] Greenhalgh, D. A., Rothnagel, J. A., Quintanilla, M. I., Orengo, C. C., Gagne, T. A., Bundman, D. S. … Roop, D. R. (1993). Induction of epidermal hyperplasia, hyperkeratosis, and papillomas in transgenic mice by a targeted v-Ha-ras oncogene. *Molecular Carcinogenesis*, *7*(2), 99–110 10.1002/mc.2940070208 7681293

[134] Brown K, Strathdee D, Bryson S, Lambie W, Balmain A (1998). The malignant capacity of skin tumours induced by expression of a mutant H-ras transgene depends on the cell type targeted. Current Biology: CB.

[135] Guasch G, Schober M, Pasolli HA, Conn EB, Polak L, Fuchs E (2007). Loss of TGFbeta signaling destabilizes homeostasis and promotes squamous cell carcinomas in stratified epithelia. Cancer Cell.

[136] Hance MW, Nolan KD, Isaacs JS (2014). The double-edged sword: conserved functions of extracellular hsp90 in wound healing and cancer. Cancers.

[137] Latil, M., Nassar, D., Beck, B., Boumahdi, S., Wang, L., Brisebarre, A., Dubois, C., Nkusi, E., Lenglez, S., Checinska, A., et al. (2017). Cell-type-specific chromatin states differentially prime squamous cell carcinoma tumor-initiating cells for epithelial to mesenchymal transition. *Cell Stem Cell*, *20*(2), 191–204195 10.1016/j.stem.2016.10.018 PMC5939571 27889319

[138] Van Keymeulen, A., Rocha, A. S., Ousset, M., Beck, B., Bouvencourt, G., Rock, J. … Blanpain, C. (2011). Distinct stem cells contribute to mammary gland development and maintenance. *Nature*, *479*(7372), 189–193 10.1038/nature10573 21983963

[139] Driskell, R. R., Lichtenberger, B. M., Hoste, E., Kretzschmar, K., Simons, B. D., Charalambous, M., Ferron, S. R., Herault, Y., Pavlovic, G., Ferguson-Smith, A. C., et al. (2013). Distinct fibroblast lineages determine dermal architecture in skin development and repair. *Nature*, *504*(7479), 277–281 10.1038/nature12783 PMC3868929 24336287

[140] Biernaskie, J., Paris, M., Morozova, O., Fagan, B. M., Marra, M., Pevny, L., & Miller, F. D. (2009). SKPs derive from hair follicle precursors and exhibit properties of adult dermal stem cells. *Cell Stem Cell*, *5*(6), 610–623 10.1016/j.stem.2009.10.019 PMC2828150 19951689

[141] Agabalyan, N. A., Rosin, N. L., Rahmani, W., & Biernaskie, J. (2017). Hair follicle dermal stem cells and skin-derived precursor cells: Exciting tools for endogenous and exogenous therapies. *Experimental Dermatology, 26*(6):505-509 10.1111/exd.13359 28418596

[142] Telerman, S. B., Rognoni, E., Sequeira, I., Pisco, A. O., Lichtenberger, B. M., Culley, O. J. … Watt, F. M. (2017). Dermal Blimp1 acts downstream of epidermal TGFβ and Wnt/β-catenin to regulate hair follicle formation and growth. *Journal of Investigative Dermatology, 137*(11), 2270-2281 10.1016/j.jid.2017.06.015 PMC5646946 28668474

[143] Yang, H., Adam, R. C., Ge, Y., Hua, Z. L., & Fuchs, E. (2017). Epithelial-mesenchymal micro-niches govern stem cell lineage choices. *Cell, 169*(3), 483-496 10.1016/j.cell.2017.03.038 PMC5510744 28413068

[144] Moulin, V., Auger, F. A., Garrel, D., & Germain, L. (2000). Role of wound healing myofibroblasts on re-epithelialization of human skin. *Burns, 26*(1), 3-12 10.1016/s0305-4179(99)00091-1 10630313

[145] Darby, I. A., Laverdet, B., Bonté, F., & Desmoulière, A. (2014). Fibroblasts and myofibroblasts in wound healing. *Clinical, Cosmetic and Investigational Dermatology ,7*, 301-311 10.2147/CCID.S50046 PMC4226391 25395868

[146] Ko, U. H., Choi, J., Choung, J., Moon, S., & Shin, J. H. (2019,). : *Physicochemically Tuned Myofbroblasts for Wound Healing Strategy*.*Scientific reports9*(*1*):*16070* 10.1038/s41598-019-52523-9 PMC6831678 31690789

[147] Green H (1991). Cultured cells for the treatment of disease. Scientific American.

